# Liver disease severity predicts carcinogenesis of dysplastic liver nodules in cirrhosis

**DOI:** 10.1038/s41598-021-00474-5

**Published:** 2021-10-25

**Authors:** Kathryn Gazelakis, Ammar Majeed, William Kemp, Bruno Di Muzio, Jan Gerstenmaier, Wa Cheung, Stuart K. Roberts

**Affiliations:** 1 Department of Gastroenterology, Alfred Health, The Alfred Hospital, 55 Commercial Rd, Melbourne, 3004 Australia; 2 Department of Radiology, Alfred Health, Melbourne, Australia; 3 Central Clinical School, Monash University, Melbourne, Australia

**Keywords:** Cancer, Gastroenterology, Risk factors

## Abstract

While dysplastic liver nodules in cirrhosis are pre-malignant, little is known about the predictors of hepatocarcinogenesis of these lesions. This was a retrospective observational study of subjects with cirrhosis who had at least one hypervascular, non-malignant intrahepatic nodule on imaging while undergoing outpatient management by a tertiary hepatology referral centre between Jan 2009 and Jan 2019. Clinical and biochemical parameters were collected. The primary endpoint was transformation to hepatocellular carcinoma (HCC) as determined by Liver Imaging Reporting and Data System. During the study period, 163 non-malignant hypervascular nodules were identified in 77 patients; 147 had at least 6 months of follow up imaging and 16 received upfront radiofrequency ablation upon detection. During a median follow up of 38.5 months (IQR 16.5–74.5), 25 (17%) of the 147 hypervascular nodules being monitored transformed to HCC. On multivariate analysis, Child–Pugh grade was found to be the only independent predictor of nodule transformation into HCC (p = 0.02). Those with Child–Pugh B and C liver disease had a 10.1 (95% CI 1.22–83.8; p = 0.03) and 32.6-fold (95% CI 2.3–467; p = 0.01) increased risk respectively for HCC transformation compared to Child–Pugh A subjects. This large, single centre study demonstrates that around 20% of dysplastic nodules in cirrhotic patients undergo hepatocarcinogenesis during follow up, and that Child Pugh grade is the only independent predictor of transformation to HCC. Additional prospective studies are warranted to better understand the risk profile of these nodules, and how best they should be managed.

## Introduction

Primary liver cancer is a leading cause of cancer-related death and the the seventh most common cancer worldwide^1–3^. Around 90% of HCC cases arise in patients with liver cirrhosis^4,5^ with the annual incidence of HCC in cirrhosis being as high as 7%^4–8^. Although the independent predictors of HCC overall are well understood, these have not been well elucidated in the literature with specific reference to a cohort of cirrhotic patients. Nevertheless, retrospective studies have identified several clinical parameters that confer an increased risk of HCC in cirrhotic liver including the presence of dysplastic nodules^8–10^.

Dysplastic nodules (DN) are pre-neoplastic lesions that occur relatively commonly in cirrhosis with the reported prevalence being 14–37% and the annual incidence being around 1.5%^11–14^. They may be defined as focal and distinctly nodular lesions without clear malignant features based on imaging and/or histopathology^11–17^. However, there is no universally accepted definition of DN in the literature while the rate of malignant transformation remains poorly understood^12–15,17^. Radiologically, DN present as arterially enhancing (i.e. hypervascular) nodules on contrast imaging with computed tomography (CT) or magnetic resonance imaging (MRI) but without the salient feature of washout during the portal venous and/or delayed phases to connote HCC^17–19^. They are however often difficult to distinguish from early HCC’s predominantly due to both being vaguely nodular and having similar histologic features with the exact point of carcinogenesis not readily identifiable^18^.

In this context, the availability of MRI with newer contrast agents such as gadoxetic acid has been a major advancement in this field by enabling a better differentiation of dysplastic nodules from early HCC^20^. Gadoxetic acid enhanced MRI was shown in a comparative 13 year systematic review and meta-analysis to have a high per-lesion sensitivity and positive predictive value of up to 85.6% and 94.2% respectively^21^. Similarly, contrast enhanced ultrasonography (CEUS) has been suggested as an option for further investigaton of indeterminate lesions^22^, having demonstrated overall per lesion sensitivity of 84.4% and positive predictive value of 89.3% for HCC^21^. Although CEUS is yet to be endorsed by current guidelines^23,24^ it can be considered as an advanced diagnostic tool in indeterminate lesions.

Currently, there is a paucity of information on the clinical and tumour risk factors for neoplastic change of premalignant lesions^9,25–29^. Small studies have suggested nodule characteristics such as size and morphology and change in size and/or vascular pattern throughout follow up are predictive, as is patient age^30–32^. In 2013 Iavarone et al.^33^ demonstrated a positive correlation toward hepatocarcinogenesis in histologically proven high grade nodules vs low grade nodules, but no significant clinical risk factors. Hence, there is insufficient published data overall to substantiate any clinical risk factors for hepatocarcinogenesis of these nodules.

In addition, there is limited data to determine how best to manage dysplastic nodules when newly identified in patients with cirrhosis^34–36^. The most widely accepted course is close surveillance of these in order to monitor for malignant transformation over time. Similarly, there is no tangible data to assess whether these nodules should be actively managed when identified such as with local thermal ablation in order to prevent hepatocarcinogenesis. Thus, we undertook this observational study to evaluate a) the epidemiology and clinical course of de novo non-malignant hypervascular nodules in patients with cirrhosis, particularly in relation to malignant transformation, and b) the baseline factors predictive of the development of HCC in hypervascular nodules detected on contrast CT or MRI.

## Methods

### Study design

This was a retrospective, single-centre, observational study of subjects with cirrhosis who were found to have at least one hypervascular, non-malignant intrahepatic nodule while undergoing management of their liver disease by the hepatology service over a 10-year period between January 31^st^ 2009 and January 31^st^ 2019. Patients were identified from electronic medical records using coding for the terms “arterially enhancing”, “dysplastic”, or “hyper-vascular” and “hepatic nodule” or “liver nodule” on radiology reports related to multiphasic contrast scans with computed tomography (CT) or magnetic resonance imaging (MRI). The protocol and any amendments received Institutional Review Board approval prior to initiation of the study with submission being undertaken via a low risk ethics application.

### Study population

To be eligible for study inclusion subjects needed to meet all the following eligibility criteria: (a) male and female patients at least 18 years of age; (b) documented history of cirrhosis; (c) presence of at least one non-malignant hypervascular intrahepatic nodule confirmed on abdominal imaging with multiphasic contrast CT and/or MRI, either at baseline or during follow up; and (d) be undergoing regular surveillance of the hypervascular nodule with imaging at 3–6 monthly intervals with at least 6 months of follow up on imaging. Subjects were excluded if they met any of the following exclusion criteria: (a) absence of cirrhosis; (b) undergone liver transplant within 6 months following detection of hyper-vascular nodule; (c) the presence of advanced HCC (macrovascular invasion and/or extrahepatic spread); and d) insufficient follow up of the nodule of < 6 months from initial detection unless treated prior. All cases with hypervascular nodules were discussed at a multi-disciplinary team meeting comprising hepatologists, diagnostic and interventional radiologists, and dedicated HCC nurse to confirm the diagnosis and determine initial management that in the vast majority of cases involved close observation and in select cases upfront ablation with RFA.

### Definitions

Cirrhosis was defined based on either (a) the combination of clinical, radiologic and laboratory criteria indicative of cirrhosis; (b) a liver stiffness measurement (LSM) > 12.5 kPa via vibration-controlled transient elastography (VCTE); or c) liver histology. Non-malignant hypervascular “dysplastic” nodules were defined on contrast-enhanced triphasic liver CT as hyperattenuating masses in the arterial phase with no washout on portal venous or delayed phases, and/or on MRI liver with primovist as the presence of a hyperattenuating mass post contrast and the absence of an increased T2 signal, restricted diffusion and washout on multiphasic post contrast imaging as described^17,37^. Nodules that met the above radiologic criteria were only considered benign and included if they had shown stability in size and appearance for at least 6 months following detection. This reflects current international guidelines on the surveillance of a liver nodule thought to be benign^36^. Past HCC was defined as evidence of HCC being definitively treated without macrovascular invasion and/or extrahepatic spread prior to detection of a benign hypervascular nodule. Current HCC was defined as evidence of a hypervascular hepatic nodule(s) with evidence of washout on CT or MRI with primovist occurring either prior to or at the time of occurrence of the non-malignant nodule being included in the study. For this study definite HCC and probable HCC were defined in cirrhotics as a lesion that was LiRADS-5 and LiRADS-4 respectively.

### Data items and extraction

Data was extracted from electronic medical records and recorded on a standardised form. Data collection included: (1) Patient characteristics: age, gender, body mass index (BMI), diabetes history; (2) Liver disease: aetiology, Child–Pugh score, Model of End-stage Liver disease (MELD) score, ALBI score; (3) Laboratory parameters: serum alpha-fetoprotein (AFP), platelet count, serum albumin, INR, creatinine, bilirubin; (4) Nodule(s) characteristics: size (mm), location, number, imaging features including LiRADS status at baseline and during follow up; (5) Treatment received to nodules; (6) HCC status: past or current; and (7) Follow up details: patient survival, HCC status, nodule status (persisted, transformed to HCC or disappeared on follow up). Transformation of nodules to HCC was determined by one of three experienced diagnostic radiologists (JG, BDM, WC) according to LiRADS criteria^38^.

### Endpoints

The primary end point of this study was the rate of malignant transformation to HCC in de novo hypervascular nodules in patients with cirrhosis. Further analysis was undertaken to determine the baseline factors predictive of the development of HCC in hypervascular nodules. The secondary endpoint was the role of radiofrequency ablation (RFA) at the time of identification of hypervascular nodules.

### Statistics

Descriptive statistics of the cohort were performed with continuous variables assessed for normality and expressed as mean ± standard deviation (SD) or median (inter-quartile range) depending on the underlying data distribution. Categorical variables were summarized using frequencies or proportions. Variables were compared using Mann–Whitney test for continuous variables and Fishers exact test for categorical ones. Univariate and multivariate stepwise Cox regression were used to evaluate the baseline factors associated with development of HCC in the nodule. As an individual patient could have more than one dysplastic nodule, the Cox model was constructed with nodules nested within individual patients, with patients treated as a random variable (frailty survival model). In addition, cumulative failure probability curves were used to examine malignant transformation of nodules among patients with cirrhosis according to baseline factors found to be significant on Cox proportional hazards analysis.

### Animal studies

This article does not contain any studies with animal subjects.

### Informed consent in human subjects

All procedures followed were in accordance with the ethical standards of the responsible committee on human The protocol and any amendments received Institutional Review Board approval prior to initiation of the study with submission being undertaken via a low risk ethics application. This included a waiver of informed consent from all patients included in the study.

### Consent to publish

The protocol and amendments received approval by The Alfred Hospital Human Research Ethics Committee prior to initiation of the study with submission being undertaken via a low risk ethics application. This include permission for publication of the data.

### Plant reproducibility

This article does not contain any studies with plants.

### Clinical trials registration

This was a retrospective cohort study that did not require clinical trials registration.

## Results

### Baseline characteristics

We identified 163 eligible nodules in 77 patients. The baseline demographic, clinical and liver disease characteristics of the cohort at the time of the index liver nodule detection are shown in Table 1. The cohort predominantly involved nodules occurring in males (n = 119, 73%) with a median age of 63.1 years (IQR 58.1–73.4). A history of diabetes present in 21.5% of subjects while the median BMI was 25.2 kg/m^2^. A current or past history of HCC was present in 24 (14.7%) and 18 (11.0%) subjects respectively, while HCV infection was the most common aetiology for cirrhosis (46.3%) followed by HBV infection (25.2%) and alcohol (6.7%). Five (3.4%) nodules occurred in patients with dual HCV and HBV aetiology, and six cases (4.1%) with mixed alcohol and HCV liver disease. Median nodule size was 10.0 mm (IQR 7.0–13.0).

**Table 1:** Baseline demographic, clinical and nodule characteristics of the overall nodule cohort and in nodules that did and did not transform to hepatocellular carcinoma.

Characteristic	Overall cohort (n = 163)	No transformation (n = 122)	Transformation (n = 25)	p value^†^
Sex: male, n (%)	119 (73)	88 (72)	16 (64)	0.47
Age (years), Median (IQR)	63.1 (58.1–73.4)	62.4 (58.1–74.5)	66.3 (57.5–71.8)	0.92
Weight (kg), Median (IQR)	78.0 (70.0–98.0)	90 (70–98.2)	75 (68–86.6)	0.25
BMI (kg/m^2^), Median (IQR)	25.2 (23.3–31.2)	27.3 (22.3–32.1)	25 (24.0–27.0)	0.97
Nodule size (mm), Median (IQR)	10.0 (7.0–13.0)	10 (7–12.5)	10 (9.0–15.5)	0.10
Past HCC, n (%)	18 (11%)	13 (10.6)	2 (8)	1.0
Current HCC, n (%)	24 (15)	12 (10)	4 (16)	0.48
**Aetiology liver disease, n (%)**				0.34
Alcohol	11 (6.7)	8 (6.6)	3 (12)	
HCV	83 (51)	56 (46)	12 (48)	
HCV and Alcohol	6 (4)	4 (3)	2 (8)	
HBV	38 (23)	33 (27)	4 (16)	
HBV and HCV	5 (3%)	3 (3)	2 (8)	
NASH	14	12 (10)	2 (8)	
NASH and Alcohol	6 (4)	6 (5)	0	
Child Pugh score^‡^, Median (IQR)	6 (5–6)	5 (5–6)	8 (6–9)	< 0.001
**Child Pugh grade**^**‡**^**, n (%)**				< 0.001
A	86 (72)	75 (83)	7 (39)	
B	27 (23)	13 (14)	7 (39)	
C	6 (5)	2	4 (22)	
MELD Score, Median (IQR)	7.0 (6.0–8.0)	6.0 (6–8)	8 (7–11)	0.002
**ALBI grade**^**‡‡**^**, n (%)**				< 0.001
1	37 (25)	33 (29)	3 (14)	
2	95 (65)	77 (68)	9 (43)	
3	14 (10)	4 (3.5)	9 (43)	
AFP level, Median (IQR)	5.6 (3.9–19.2)	6.1 (4–11.9)	4.7 (2.7–41)	0.98
Diabetes, n (%)	35	25 (21)	8 (32)	0.29

AFP, alpha-fetoprotein; ALBI, albumin-bilirubin grade for hepatocellular carcinoma; BMI, body.

mass index; HCC, hepatocellular carcinoma; HBV, hepatitis B virus; HCV, hepatitis C virus;

MELD, model for end-stage liver disease; NASH, non-alcoholic steatohepatitis.

^‡^in 44 (27%) nodules the result was not available.

^‡‡^in 17 (10.4%) of nodules the result was not available.

^†^Comparison of nodules that transformed to HCC versus nodules that did not.

### Initial management

Sixteen (10%) of the 163 hypervascular nodules underwent definitive initial treatment with RFA. There were key differences between subjects with nodules that did and did not receive RFA at detection (see supplementary Table 1). In particular, subjects receiving RFA upfront were more likely to have a current history of HCC at a different site to the studied nodule compared to those not receiving RFA (50% vs 11% respectively; p < 0.001). In addition, Child–Pugh Score (p = 0.01) and Grade (p = 0.01) were both significantly higher in the RFA group compared to the non-RFA group. Interestingly, those receiving RFA had a smaller median nodule size compared to the non-RFA group (7 mm vs 10 mm respectively; p = 0.05). None of the patients receiving upfront RFA to dysplastic nodules developed HCC at the ablation zone.

### HCC transformation during follow up

**Figure 1:**
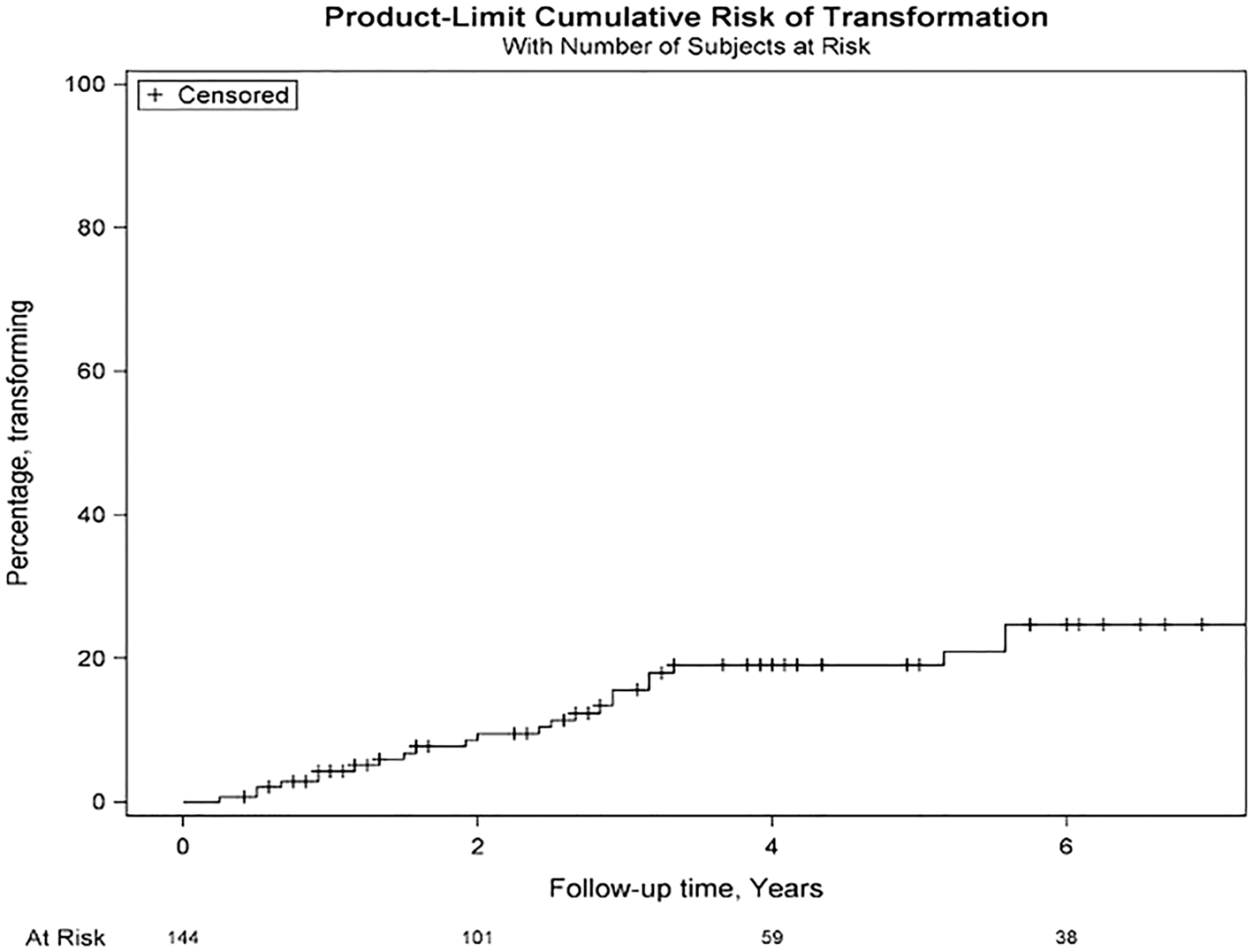
Cumulative probability curve of transformation of hypervascular nodules to hepatocellular carcinoma (entire cohort).

During a median follow up of 38.5 months (IQR 16.5–74.5), 25 (17%) of the 147 hypervascular nodules not receiving initial RFA transformed to HCC. The median time to transformation was 29.0 months (IQR 12.5–38.0 months). The 1-, 2-, and 3-year rate of transformation of dysplastic nodules to HCC was 4.3%, 9.5% and 15.7% respectively. The cumulative probability of progression to HCC of the overall cohort is shown in Fig. 1. The percentage of nodules progressing to HCC by Child–Pugh Grade is shown in Table 2. Interestingly, nodules occurring in Child–Pugh C patients transformed to HCC at a rate of 20% at 1 year, and 47% and 73% at 2 and 3 years respectively with the risk of transformation reducing among those with lower Child–Pugh status.

**Table 2:** Rate of hepatocarcinogenesis of dysplastic nodules by Child–Pugh grade.

Time (years)	Transformation (%)		
	Child–Pugh A	Child–Pugh B	Child–Pugh C
1	1	10	20
2	4	16	47
3	8	38	73

### Baseline characteristics according to HCC transformation

The baseline clinical and liver disease characteristics of subjects according to whether transformation to HCC occurred over time or not are shown in Table 1. Of note, the median age and gender of patients at the time of nodule diagnosis were similar between those with nodules that did and did not transform. There was no difference in the distribution of aetiologies of cirrhosis between the two groups while median nodule size was also similar in both groups at 10 mm. In comparison, the median MELD at the time of nodule diagnosis was higher in those with nodules that transformed compared to those that did not (8 vs 6; p = 0.002).

### Predictors of HCC transformation

**Figure 2:**
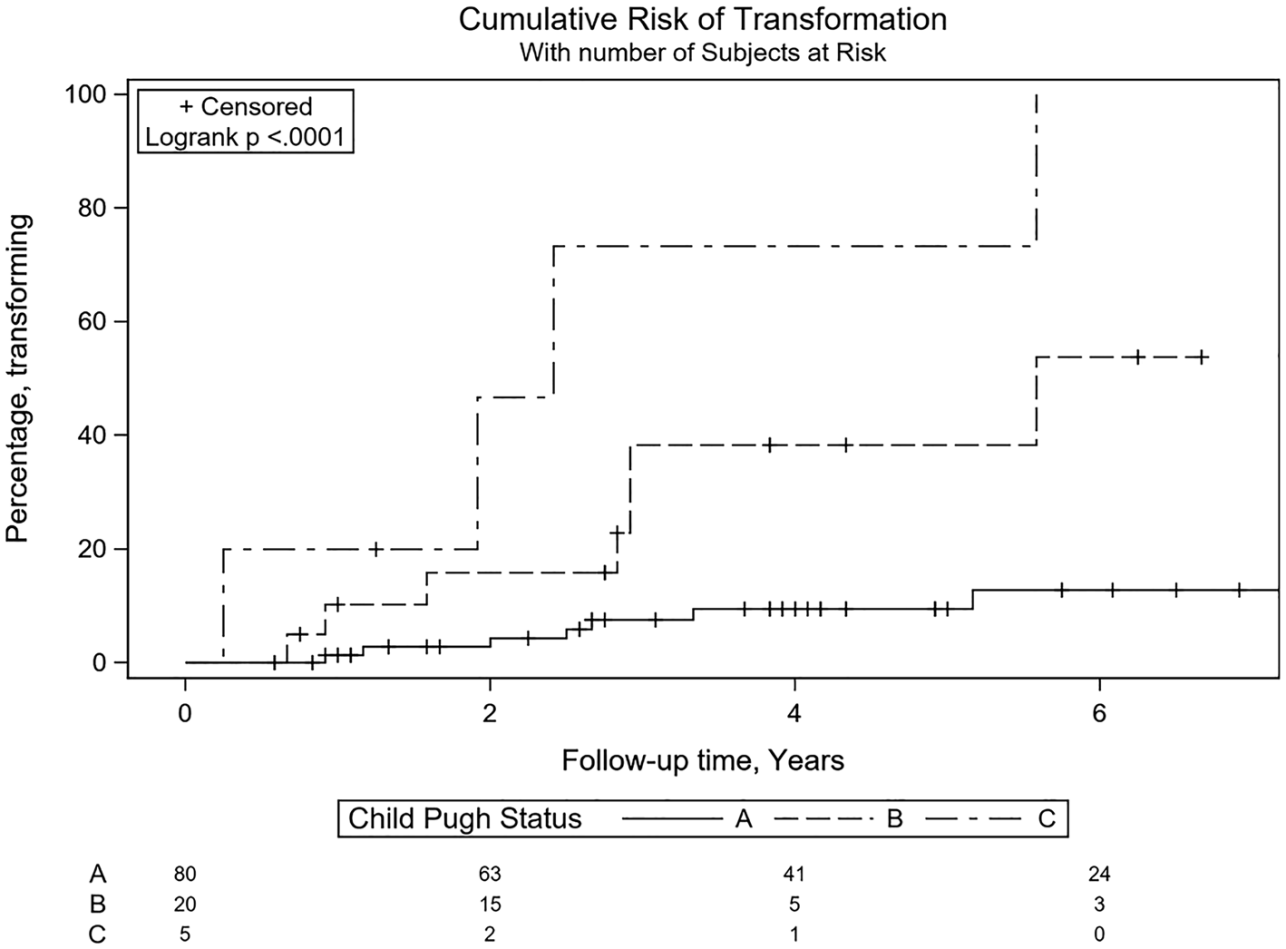
Cumulative probability curve of transformation of hypervascular nodules to hepatocellular carcinoma according to Child–Pugh grade.

On univariate analysis, dysplastic nodule transformation to HCC was associated with Child–Pugh score (p < 0.001) and grade (p < 0.001), MELD (p = 0.002) and ALBI scores (p < 0.001). Notably, median AFP level at nodule diagnosis was not associated with malignant transformation (6.1 vs 4.7; p = 0.94). Other clinical parameters not significant on univariate analysis were age, sex, BMI, aetiology of liver disease, size of lesion, HCC status, and diabetes (Table 3). On multivariate analysis, Child–Pugh grade was found to be the only independent predictor of nodule transformation into HCC (p = 0.02). Notably, those with Child–Pugh B and C liver disease had a 10.1 (95% CI 1.22–83.8; p = 0.03) and 32.6-fold (95% CI 2.3–467; p = 0.01) increased risk respectively for HCC transformation over time compared to Child–Pugh A subjects (Table 3, Fig. 2).

**Table 3:** Univariate and multivariate analysis of baseline demographic, clinical and nodule characteristics associated with malignant transformation of nodules to hepatocellular carcinoma.

Variable	Univariate analysis			Multivariate analysis		
	Hazard ratio*	95% CI	P	Hazard Ratio**	95% CI**	P
Gender: male	1.05	0.46–2.39	0.91	3.71	0.34–40.93	0.28
Age (years)	0.99	0.96–1.02	0.39	1.02	0.94–1.11	0.66
Age > 60 years	1.22	0.51–2.92	0.66			
Weight (kg)	1.00	0.97–1.02	0.68			
BMI (kg/m^2^)	1.03	0.97–1.09	0.38			
Diabetes	1.83	0.78–4.27	0.17			
Past HCC	0.62	0.14–2.66	0.52			
Current HCC	1.42	0.48–4.19	0.53			
Nodule size (mm)	1.01	0.95–1.08	0.77			
AFP (ng/ml)	1.00	1.00–1.00	0.94			
Child–Pugh score (per unit increase)	1.83	1.45–2.30	** < 0.001**			
**Child Pugh status**						
A (reference)	–					
B	4.63	1.62–13.24	**0.004**	10.1	1.22–84	**0.03**
C	16.2	4.66–56	** < 0.001**	32.6	2.27–467	**0.01**
MELD (per unit increase)	1.14	1.06–1.24	** < 0.001**			
**ALBI grade**						
1 (reference)	–					
2	1.26	0.34–4.65	0.73			
3	13.5	3.63–50	** < 0.001**			

*Univariate analysis using Cox regression.

**Multivariate Cox model adjusting for age, sex and Child–Pugh Grade.

Bold significance P < 0.05.

### Outcome of non-transforming nodules

Of the 122 nodules that did not transform, 47 (38.5%) spontaneously disappeared with a median time to disappearance of 31.0 months (IQR 12.0–36.5) while 75 (61.5%) remained stable, over a median follow up time of 59.0 months (IQR 33.0–95.0). Although the median MELD score was not dissimilar between the two groups (median 6 vs 8 respectively), nodules were more likely to disappear or remain stable throughout follow up with a lower MELD score (p = 0.02). Similarly, a lower ALBI Grade conferred a higher chance of spontaneous disappearance or stability of a nodule (p = < 0.001).

## Discussion

In this real-world observational study, we evaluated the clinical course of de novo non-malignant hypervascular nodules in patients with cirrhosis particularly in relation to malignant transformation to HCC. Our clinical experience demonstrates that around one-fifth of these nodules transformed into HCC over time with the 1-, 2-, and 3-year rate of dysplastic nodule transformation to HCC being 4.3%, 9.5% and 15.7% respectively. In addition, we found that Child Pugh grade was the only independent predictor of nodule transformation into HCC (p = 0.02).

In our cohort, we found that 17% of dysplastic nodules that did not undergo RFA at detection transformed into HCC over a median follow up time of 38.5 months with the median time to hepatocarcinogenesis being 28 months. Given this represented 15.3% of the entire cohort, the results are similar to those of Kobayashi et al.^25^ who reported an overall HCC transformation rate of 18.8% over a median follow up time of 33.6 months. Similarly, Sato et al.^27^ found that 13.6% of dysplastic nodules transformed over a median follow up time of 31.9 months, while Seki et al.^29^ a rate of 12.5% over a median follow up period of 25 months. Our annual rate of HCC transformation of around 5% was lower than that of Iavarone et al.^28^ who reported a yearly rate of 9.2% over a similar follow up period of 39 months. Thus, it appears from published data that the annual incidence of HCC from these hypervascular at-risk nodules is somewhere between 5 and 9%.

Currently there is a paucity of information in the literature on the clinical and biochemical parameters influencing transformation of benign hypervascular nodules to HCC^26–28,30–33^. In our study, we found that Child–Pugh grade was the only independent predictor of hepatocarcinogenesis on multivariate analysis although other markers of liver disease severity including MELD and ALBI were associated with HCC transformation on univariate analysis. Indeed, the risk of developing HCC in those with Child–Pugh C cirrhosis was around 30-fold higher compared to those with compensated Child–Pugh A cirrhosis. Moreover, Child–Pugh grade correlated with higher rates of hepatocarcinogenesis (Table 3). This is not surprising given higher Child–Pugh scores and grade portend to a greater risk of HCC in cirrhotic patients and hence in this study, predict progression to HCC with time elapsing from initial detection. Notably, MELD score as another marker of liver disease severity was also a predictor on univariate analysis but did not remain so on multivariate analysis.

In contrast, Kobayashi et al.^25^ demonstrated that age > 60, tumour diameter, and detection on helical CT were significant predictors of transformation but found no significant association with aetiology of liver disease, AFP level, serum platelet, count, and serum prothrombin time. In addition, other literature utilising liver histopathology has depicted older age and high-grade histology as being significant factors in malignant transformation^28^. It is well established that clinical prognostic factors for HCC in cirrhosis include serum bilirubin and albumin levels, clinically-significant portal hypertension, ascites and functional status^39–42^. Thus, it is not surprising that Child–Pugh status remained a significant predictor for malignant transformation on multivariate analysis. This finding is potentially important from a prognostic and clinical management perspective, particularly for Child–Pugh B and C patients, since current guidelines recommend against routine HCC surveillance in Child–Pugh C patients, unless they are on a liver transplant waiting list^23,24^. However, in clinical practice dysplastic nodules are often followed up with 3–6 monthly imaging irrespective of Child–Pugh status. Still, larger, prospective studies are required to more accurately define the clinical predictors of hepatocarcinogenesis in cirrhotic patients with dysplastic liver nodules and the appropriate surveillance strategy during follow up.

Another important finding of our study that is relatively under-appreciated in the literature is the disappearance on imaging of these seemingly “at-risk” nodules. We found that 38.5% of nodules disappeared on follow up ultrasound or CT with a median time to disappearance of 31 months. This is similar to that of Sato et al.^27^ who found 40% disappear at 50 months, while Seki et al.^29^ reported a 46% disappearance rate over a median follow up of 25 months. In contrast Kobayashi et al.^25^ found only one of 154 (0.7%) of nodules disappear during follow up. While we did find an association between ALBI grade and nodule disappearance, currently there are no reliable predictor(s) of nodule disappearance, so consequently, all need follow-up imaging over time to monitor for malignant transformation.

To date, there is no consensus on the optimal management of hypervascular pre-malignant liver nodules when detected *vis a vis* definitive management versus watchful observation^34,36,40^. While it appears intuitively obvious to ablate high-risk lesions when identified, Cho et al.^34^ found no distinct survival benefit of RFA of high grade dysplastic nodules that would support such an approach. In our cohort, sixteen hepatic nodules underwent definitive thermal ablation upon detection, many of whom occurred in patients with an active history of HCC at a separate site. During a median follow up of 13 months, none of nodules that were initially treated with RFA developed HCC at the ablation zone. However, eight (50%) of the nodules occurred in five (31%) individual patients with active HCC at a separate site and ten (62.5%) nodules occurred in patients with either past or current HCC. This highlights their high-risk status. In the absence of past HCC, upfront thermal ablation of an indeterminate or suspicious nodule could be curative for some patients and prevent the development of HCC. However, this invasive therapy must be carefully weighed up against its potential risks and there is currently no role for thermal ablation in patients with no past history of HCC. Clearly, more robust data is needed to guide clinical management in this space, and specifically, taking into account clinical risk factors of Child–Pugh status and past or current history of HCC. There is a randomised clinical trial underway to address the role of active therapy of dysplastic nodules^43^.

The strengths of this study include its sizeable cohort, well-established criteria for benign nodule inclusion and HCC transformation, independent radiological review using LiRADS to enhance diagnostic accuracy of HCC transformation, management of nodules via a multi-disciplinary team and appreciable follow up period that enabled a large number of nodules to be captured and assessed. In addition, it assessed the clinical and biochemical parameters influencing HCC transformation in a real-world context. However, there were important limitations to the study notwithstanding it’s retrospective design. This included the requirement for data retrieval on nodules, cirrhosis and hepatocellular carcinoma status from electronic medical records as opposed to predetermined criteria for the purposes of the study. In addition, there were no pre-defined protocolized criteria for management of these nodules in terms of ablation versus surveillance including monitoring frequency, although in all cases it was based on the opinion of the multi-disciplinary team managing these patients. Finally, being retrospective not all data was complete on clinical and biochemical parameters.

In conclusion, this study has demonstrated for the first time a strong relationship between liver disease severity and hepatocarcinogenesis of dysplastic/ hypervascular liver nodules in patients with cirrhosis. Given the absence of clinical practice guidelines to inform clinicians of how dysplastic nodules should be managed, our findings highlight an urgent need for prospective studies of these nodules including randomised clinical trials particularly in those with advanced liver disease to determine the most appropriate course of action when they are detected.

## Supplementary Information

Supplementary Information.

## Data Availability

Data will be made available upon request related to study findings.

## Abbreviations

DN: Dysplastic nodules
CT: Computed tomography
HCC: Hepatocellular carcinoma
LiRADS: Liver Imaging Reporting and Data System
MRI: Magnetic resonance imaging
RFA: Radiofrequency ablation
CEUS: Contrast enhanced ultrasound
