# Supplementary material for: Liver disease severity predicts carcinogenesis of dysplastic liver nodules in cirrhosis
Source: Sci Rep. 2021 Oct 25;11:20954. doi: 10.1038/s41598-021-00474-5 (PMC8545953; doi:10.1038/s41598-021-00474-5)
Supplement: Supplementary file 1 — Supplementary Information. [file 41598_2021_474_MOESM1_ESM.docx]

# **Supplementary Appendix**

**Table 1**. Comparison of nodules undergoing upfront radiofrequency ablation vs those that

did not

| Characteristic* | No RFA  (N=147) | RFA  (N=16) | p-value^†^** |
| --- | --- | --- | --- |
| Sex: male, n (%) | 104 (70.8) | 15 (93.8) | 0.07 |
| Age (years), (IQR) | 62.9 (58.1-73.5) | 65.41(62.9-65.6) | 0.89 |
| Nodule Size (mm), Median (IQR) | 10 (7-13) | 7 (3-11) | 0.05 |
| Weight (kg), Median (IQR) | 80.9 (68-98) | 78 (72-79) | 0.44 |
| BMI (kg/m^2^), Median (IQR) | 25.8 (22.9-31.4) | 24.1 (23.8-27.3) | 0.37 |
| MELD score, Median (IQR) | 7 (6-8) | 8 (6-10) | 0.17 |
| AFP level (ng/mL), Median (IQR) | 5.5 (4.0-14.5) | 19.3 (2.5-60.0) | 0.23 |
| Past HCC, n (%) | 15 (10.2) | 3 (18.3) | 0.39 |
| Current HCC, n (%) | 16 (10.9) | 8 (50) | <0.001 |
| Aetiology liver disease, n (%) |  |  |  |
| Alcohol | 11 (7.5) | 0 (0) | 0.10 |
| HBV | 37 (25.2) | 1 (6.3) |  |
| HBV & HCV | 5 (3.4) | 0 (0) |  |
| HCV | 68 (46.3) | 15 (93.8) |  |
| HCV & ETOH | 6 (4.1) | 0 (0) |  |
| NASH | 14 (9.5) | 0 (0) |  |
| NASH & ETOH | 6 (4.1) | 0 (0) |  |
| Child-Pugh score, n (%) |  |  |  |
| 5 | 51 (47.2) | 4 (36.4) | 0.01 |
| 6 | 31 (28.7) | 0 (0) |  |
| 7 | 11 (10.2) | 6 (54.6) |  |
| 8 | 3 (2.8) | 1 (9.1) |  |
| 9 | 6 (5.6) | 0 (0) |  |
| 10 | 4 (3.7) | 0 (0) |  |
| 11 | 2 (1.9) | 0 (0) |  |
| Child- Pugh Grade, n (%): |  |  |  |
| A | 82 (75.9) | 4 (36.4) | 0.01 |
| B | 20 (18.5) | 7 (63.6) |  |
| C | 6 (5.6) | 0 (0) |  |
| Diabetes, n (%) | 33 (22.5) | 2 (12.5) | 0.53 |
| ALBI Grade, n (%): |  |  |  |
| 1 | 36 (26.7) | 1 (9.1) | 0.37 |
| 2 | 86 (63.7) | 9 (81.8) |  |
| 3 | 13 (9.6) | 1 (9.1) |  |
| Alcohol History, n (%) | 47 (32.0) | 1 (6.3) | 0.04 |

AFP, alpha-fetoprotein; ALBI, albumin-bilirubin grade for hepatocellular carcinoma; BMI, body

mass index; HCC, hepatocellular carcinoma; HBV, hepatitis B virus; HCV, hepatitis C virus;

MELD, model for end-stage liver disease; NASH, non-alcoholic steatohepatitis; RFA, Radiofrequency Ablation

*Data presented as median (IQR) unless stated otherwise

** Mann–Whitney U test for continuous variables or Fisher's exact test for categorical variables

^†^ Comparison of nodules that received RFA versus those that did not
